# Risk of emergency surgery for complicated appendicitis: Japanese nationwide study

**DOI:** 10.1002/ags3.12408

**Published:** 2020-11-09

**Authors:** Takeshi Yamada, Hideki Endo, Hiroshi Hasegawa, Toshimoto Kimura, Yoshihiro Kakeji, Keiji Koda, Hideyuki Ishida, Kazuhiro Sakamoto, Keiji Hirata, Hiroyuki Yamamoto, Hiroaki Miyata, Akihisa Matsuda, Hiroshi Yoshida, Yuko Kitagawa

**Affiliations:** ^1^ Department of Gastrointestinal and Hepato‐Biliary‐Pancreatic Surgery Nippon Medical School Tokyo Japan; ^2^ Department of Healthcare Quality Assessment Graduate School of Medicine The University of Tokyo Tokyo Japan; ^3^ Project Management Subcommittee The Japanese Society of Gastroenterological Surgery Tokyo Japan; ^4^ Database Committee The Japanese Society of Gastroenterological Surgery Tokyo Japan; ^5^ Department of Surgery Teikyo University Chiba Comprehensive Medical Center Chiba Japan; ^6^ Department of Digestive Tract and General Surgery Saitama Medical University Saitama Japan; ^7^ Department of Coloproctological Surgery Juntendo University Faculty of Medicine Tokyo Japan; ^8^ Department of Surgery 1 University of Occupational and Environmental Health Kitakyushu Japan; ^9^ The Japanese Society of Gastroenterological Surgery Tokyo Japan

**Keywords:** appendicitis, complicated appendicitis, morbidity, mortality, uncomplicated appendicitis

## Abstract

**Aim:**

Appendicitis is divided into two categories: complicated appendicitis (CA) and uncomplicated appendicitis (UA). In pediatric patients with CA, the use of interval appendectomy (IA), which is non‐operative management followed by elective surgery, has decreased the number of postoperative complications. Before discussing the merit of IA for adult patients, we need to clarify whether the frequency and seriousness of the complication rate after emergency surgery is higher for CA than for UA.

**Methods:**

This retrospective cohort study included adult patients who underwent appendectomy and who were registered in the National Clinical Database (NCD) from 2014 to 2016. Patients with CA who underwent emergency appendectomy comprised the CA group. Patients with UA comprised the UA group. Patients with chronic or recurrent appendicitis who underwent elective appendectomy comprised the elective appendectomy (EA) group. Primary outcomes were all morbidity, serious morbidity, and mortality within 30 days after appendectomy.

**Results:**

We included 109 256 patients in the study: 14 798 CA, 86 876 UA, and 7582 EA patients. Compared with the UA group, the rates of all morbidity, serious morbidity, and mortality were significantly higher in the CA group. All morbidity, serious morbidity, and mortality rates were significantly lower in the EA group than in the other two groups.

**Conclusions:**

We confirmed that emergency surgery for CA places the patient at relatively higher risk. We also showed that the risk associated with EA is significantly lower than that for the other methods.

## Graphical abstract

This retrospective cohort study included adult patients who underwent appendectomy and who were registered in the National Clinical Database from 2014 to 2016. Compared with the uncomplicated appendicitis group, the rates of all morbidity, serious morbidity, and mortality were significantly higher in the complicated appendicitis group. All morbidity, serious morbidity, and mortality rates were significantly lower in the elective appendectomy group than in the other two groups.

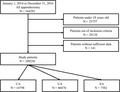

## 1 INTRODUCTION

Acute appendicitis is one of the most common causes of an acute abdomen and is

the disease that most commonly requires surgery. Emergency appendectomy has been the standard of care for treating acute appendicitis. Nevertheless, as early as 1946 it was recognized that non‐operative management (antibiotics) can sometimes be used to successfully treat appendicitis.^1^ Recently, appendicitis has been divided into two categories: complicated appendicitis (CA), which indicates the presence of a peritoneal abscess, and uncomplicated appendicitis (UA), which does not have a peritoneal abscess. In pediatric patients with CA, the use of interval appendectomy (IA), which is non‐operative management followed by elective surgery, has decreased the number of postoperative complications.^2^ Unlike appendicitis with pan‐peritonitis, which necessitates emergency surgery, non‐operative management that includes computed tomography‐guided drainage is acceptable for treating CA because its success rate is high.

There have been warnings raised, however, against the use of non‐operative management and IA for CA. In one study, post‐intervention peritonitis was significantly more frequent in the non‐operative management group than in the emergency surgery group.^3^ In another, the complication‐free rate was higher for emergency surgery of CA than that for non‐operative management.^4^ In addition, approximately 30% of the patients underwent surgery within 1 year after successful non‐operative management.^3^, ^5^ The most common objection to IA has been that it is unnecessary because the morbidity rate following emergency surgery for CA was within an acceptable range.^6^, ^7^, ^8^

Before discussing the merit of IA, we need to clarify whether the frequency and seriousness of the complication rate after emergency surgery is higher for CA than for UA. If the outcome of emergency surgery for CA is equal to that for UA, the usefulness of carrying out IA is limited. If the outcome of emergency surgery for CA is not acceptable, we must develop a better treatment, including IA.

We therefore compared morbidity and mortality rates associated with emergency surgery for CA versus those for UA and elective surgery, obtaining the data from a Japanese nationwide database (National Clinical Database; NCD).

## 2 METHODS

### 2.1 National Clinical Database

The NCD in Japan, initiated in 2011, was developed in collaboration with the American College of Surgeons National Surgical Quality Improvement Program (ACS NSQIP) in the USA with a shared goal of creating a standardized surgery database for quality improvement. The NCD and the ACS NSQIP have developed systems using standardized definitions of variables to collect data relating to risk factors and outcomes. The NCD is a nationwide web‐based data‐entry system which is linked to the surgical board certification system in Japan. Patient data are registered only in the NCD system (http://www.ncd.or.jp/), which is operated by the Japan Surgical Society. The NCD now covers more than 97% of all surgical procedures in Japan. Currently, 1 200 000 patients who undergo surgery annually are registered from approximately 5000 institutions.^9^

### 2.2 Study design

This retrospective cohort study included adult patients (aged ≥ 18 years) who underwent appendectomy and who were registered in the NCD from 1 January, 2014 to 31 December, 2016. The clinical data and surgical outcome for this study were obtained from the NCD. Use of data from the registry for retrospective observational studies was approved by the institutional review board of the Japanese Society for Abdominal Emergency Medicine and the institutional review board of Nippon Medical School. This study was supported by a grant from the Japanese Society for Abdominal Emergency Medicine.

Patients who met the criteria of K35.1 (acute appendicitis with peritoneal abscess) of the World Health Organization’s International Statistical Classification of Diseases and Related Health Problems, 10th revision and underwent emergency appendectomy for CA comprised the CA group. Patients who met the criteria of K35.9 (acute appendicitis without generalized peritonitis, perforation, peritoneal abscess, or rupture) appendectomy comprised the UA group. Patients who met the criteria of K36 (other [chronic, recurrent] appendicitis) and underwent elective appendectomy comprised the elective appendectomy (EA) group. We excluded patients who met the criteria of K36 and underwent emergency appendectomy, and patients who met the criteria of K35.1 and underwent elective appendectomy.

Primary outcomes were all morbidity, moderate morbidity, serious morbidity, and mortality within 30 days after appendectomy. Secondary outcomes included hospital length of stay (LOS), rate of readmission within 30 days after surgery, and laparoscopic surgery rate. Overall morbidity was defined as having documentation of a Clavien‐Dindo grade ≥ I complication, moderate morbidity as grade ≥ II and serious morbidity as grade ≥ III. Clavien‐Dindo grades^10^ are shown in Table 1.

**Table 1:** Clavien‐Dindo grades

Grade	
I	Any deviation from the normal postoperative course without the need for pharmacological treatment or surgical, endoscopic, and radiological interventions. Allowed therapeutic regimens are as follows: drugs as antiemetics, antipyretics, analgesics, diuretics, electrolytes, and physiotherapy. This grade also includes wound infections opened at the bedside
II	Requiring pharmacological treatment with drugs other than those allowed for grade I complications. Blood transfusions and total parenteral nutrition are also included.
III	Requiring surgical, endoscopic or radiological intervention
IV	Life‐threatening complications (including CNS complications) requiring intermediate care/intensive care unit management
V	Death of a patient

Abbreviation: CNS, central nervous system.

### 2.3 Statistical analysis

Age, operation duration, blood loss, and LOS were analyzed using the Kruskal‐Wallis method. Gender, surgical method (laparoscopic or open surgery), American Society for Anesthesiologists (ASA) physical status classification, morbidity, mortality, and 30‐day readmission were analyzed using the χ^2^ test. All statistical analyses were carried out using R software. (version 3.5.0, 2018; R Foundation for Statistical Computing, Vienna, Austria).

## 3 RESULTS

### 3.1 Patients

**Figure 1:**
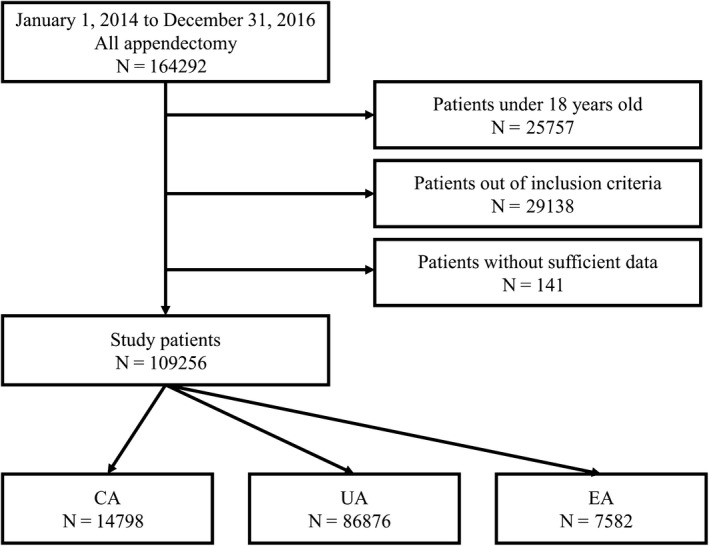
Flow diagram. Between 2014 and 2016, 164 292 patients who underwent appendectomy were registered in the National Clinical Database (NCD). Patients <18 y (n = 25 757), without inclusion criteria (n = 29 138), or without sufficient data (n = 141) were excluded, leaving 109 256 patients in the study: 14 798 complicated appendicitis (CA), 86 876 uncomplicated appendicitis (UA), and 7582 patients with elective appendectomy (EA)

Between 2014 and 2016, 164 292 patients who underwent appendectomy were registered in the NCD. Patients <18 years (n = 25 757), without inclusion criteria (n = 29 138), or without sufficient data (n = 141) were excluded, leaving 109 256 patients in the study: 14 798 CA, 86 876 UA, and 7582 EA patients (Figure 1). Median age, proportion of men, and proportion of ASA 3 or 4 were higher in the CA group than in the UA and EA groups. The open surgery rate was significantly higher in the CA group (45.5%) than in the UA (36.0%) and EA (12.3%) groups (Table 2).

**Table 2:** Patients’ background and surgical outcomes

	Complicated appendicitis (CA) N = 14 798	Uncomplicated appendicitis (UA) N = 86 876	Elective appendectomy (EA) N = 7582	*P* value
Patients’ background				
Age, y (median [IQR])	52 [38, 68]	41 [29, 57]	45 [32, 62]	<.001
Male (%)	8792 (59.4)	48 280 (55.6)	3741 (49.3)	<.001
ASA 1 (%)	7860 (53.1)	56 288 (64.8)	4888 (64.5)	<.001
ASA 2 (%)	5951 (40.2)	27 737 (31.9)	2478 (32.7)	
ASA 3 (%)	933 (6.3)	2701 (3.1)	212 (2.8)	
ASA 4 (%)	47 (0.3)	109 (0.1)	1 (0.0)	
ASA 5 (%)	7 (0.05)	41 (0.05)	3 (0.04)	
Surgical outcome				
Laparoscopic (%)	8067 (54.5)	55 607 (64.0)	6647 (87.7)	<.001
Surgical time (min) (median [IQR])	77 [57, 105]	60 [44, 81]	62 [45, 90]	<.001
Blood loss (mL) (median [IQR])	10 [1, 42]	5 [0, 10]	2 [0, 5]	<.001
LOS (day) (median [IQR])	9.00 [6.00, 12.00]	6.00 [4.00, 8.00]	6.00 [5.99, 7.00]	<.001
Morbidity				
≥C‐D Grade I (%)	2938 (19.9)	6322 (7.3)	355 (4.7)	<.001
≥C‐D Grade II (%)	1584 (10.7)	2998 (3.5)	150 (2.0)	<.001
≥C‐D Grade III (%)	448 (3.0)	702 (0.8)	33 (0.4)	<.001
Readmission (%)	344 (2.3)	978 (1.1)	56 (0.7)	<.001
Mortality (%)	19 (0.13)	25 (0.04)	2 (0.03)	<.001

Abbreviations: ASA, American Society of Anesthesiologists physical status classification; C‐D, Clavien‐Dindo classification; IQR, interquartile range; LOS, hospital length of stay.

### 3.2 Surgical outcome, mortality, complications

Operative duration was longer for the CA group than for the UA and EA groups, and blood loss was greater in the CA group than in the UA and EA groups. LOS was longer in the CA group than in the UA and EA groups. Among all patients, 9615 (8.8%) experienced morbidity, and 46 (0.04%) died. Rates of all morbidity, moderate morbidity, serious morbidity, readmissions, and mortality in the CA group were significantly higher than in the other two groups. All morbidity, serious morbidity, readmission, and mortality rate were significantly lower in the EA group than in the other two groups.

## 4 DISCUSSION

This nationwide database analysis, which included 109 256 patients with appendicitis, examined the incidence of 30‐day overall morbidity, serious morbidity, and mortality after appendectomy by emergency surgery for CA and UA and elective surgery for appendicitis at NCD‐participating hospitals. This analysis confirmed that, although patients undergoing appendectomy for acute appendicitis represent a relatively low‐risk population, significantly greater risk is associated with emergency surgery for CA. It has been believed that delay in surgical removal of a diseased appendix can result in a poor outcome and that emergency appendectomy is safe. Thus, emergency appendectomy is the gold standard for treating both UA and CA. The results of the present study, however, raise serious questions about the safety of an emergency appendectomy for CA in terms of morbidity and mortality. The same results were reported in a large‐cohort, prospective study^11^ and in another, nationwide study.^12^, ^13^ The authors of those studies concluded that the ideal treatment for CA may be different from that for UA.

Using our latest nationwide database, we confirmed that emergency surgery for CA poses a greater risk than that of UA. Several studies based on nationwide databases reported that the mortality rates associated with appendectomy range from 0.04% to 0.24%^12^, ^13^, ^14^, ^15^, ^16^, ^17^, ^18^, ^19^, ^20^, ^21^, ^22^ (Table 3), similar to our results for the UA patients. The mortality rate for CA patients in the present study (0.13%), which was lower than that for CA patients in a nationwide study in the USA (0.18%–0.30%),^12^, ^13^ was significantly higher than that for the UA and EA patients. Nationwide studies in the USA and Finland reported that CA places patients at risk of death.^12^, ^13^, ^18^ Considering that the present study did not include appendicitis associated with peritonitis, the high mortality rate associated with CA must be verified.

**Table 3:** Nationwide study of appendicitis

Author	Published	Database	Country	Duration	No. patients	Morbidity All patients	Mortality All patients	Morbidity CA	Mortality CA
Guller^14^	2004	NIS	USA	1997	43 757	10.66	0.24	NA	NA
Ingraham^12^	2010	ACS NSQIP	USA	2005‐2008	32 683	5.50	0.09	14.00	0.30
Yeh^15^	2011	National Health Research Institutes	Taiwan	2001‐2008	166 690	NA	0.04	NA	NA
Masoomi^13^	2011	NIS	USA	2006‐2008	573 244	4.80	0.04	22.71	0.18
Andersson^16^	2014	National Patient Register	Sweden	1992‐2008	169 896	12.72		NA	NA
Ceresoli^17^	2016	Bergamo's district	Italy	1997‐2013	16 544	NA	0.05	NA	NA
Kotaluoto^18^	2017	Hospital Discharge Register	Finland	1990‐2010	164 579	NA	0.21	NA	NA
Alore^19^	2018	ACS NSQIP	USA	2012‐2015	112 122	6.40	0.09	NA	NA
Horn^20^	2018	NIS	USA	2010‐2014	131 162	NA	0.08	NA	NA
Sartelli^21^	2018	POSAW	Worldwide	2016	4282	9.20	0.28	NA	NA
Canal^22^	2020	AQC	Switzerland	2010‐2017	9224	4.67	0.12	NA	NA
Yamada (present study)	2020	NCD	Japan	2014‐2016	109 256	8.80	0.04	19.90	0.13

Abbreviations: ACS NSQIP, The American College of Surgeons National Surgical Quality Improvement Program; AQC, Arbeitsgemeinschaft für Qualitätssicherung in der Chirurgie [Working Group for Quality Assurance in the Surgical Disciplines]; NA, not available; NCD, National Clinical Database; NIS, Nationwide Inpatient Sample; POSAW, Prospective Observational Study on acute Appendicitis Worldwide.

Because of its morbidity, the safety of emergency surgery for CA is also open to question. The incidence of all morbidity was 19.9% and that of serious morbidity for CA patients was 3.0%. The overall morbidity rates for CA patients were reported at 14.0% in the ACS NSQIP database and 22.71% in the National (Nationwide) Inpatient Sample database. In the MUSTANG study, the morbidity rate for UA was 5% and that for perforated appendicitis was 32%. Moreover, secondary interventions were needed for 1% of UA patients and for 6% of CA patients.^11^ A Polish and German multi‐center study also showed that CA is a risk factor for serious morbidity (Clavien‐Dindo grades III‐IV.^23^ The incidence of grade ≥ II morbidity after CA was threefold higher than that after UA, fivefold higher than that after EA, and nearly equal to the incidence after distal gastrectomy (11.5%)^24^ or right hemicolectomy (13.3%)^25^ reported by the NCD. Notably, the all‐morbidity and serious‐morbidity rates were much lower in EA patients than in UA and CA patients. These facts indicate that CA may need a strategy different from that used for UA to decrease mortality and morbidity.

Open surgery was carried out in 45% of the CA patients, which is a significantly higher rate than that for UA or EA patients. Recent studies show that laparoscopic appendectomy provides considerable benefits over open appendectomy, including a lower morbidity rate,^26^, ^27^, ^28^ a shorter LOS,^27^, ^28^, ^29^ a lower rate of small bowel obstruction,^30^ less postoperative pain,^27^, ^28^ and earlier postoperative recovery.^28^, ^29^ These facts indicate problems with the use of emergency surgery for CA. The longer operative duration is also a negative factor for choosing emergency surgery for patients with CA.

Although it is generally assumed that untreated appendicitis eventually perforates, epidemiological differences between CA and UA in the present and other reported studies indicated that CA and UA may present with different states of appendicitis. In the present study, CA patients were older and at a higher ASA class than the UA patients, as has been reported in other large studies,^11^, ^31^ a nationwide study,^32^ and a meta‐analysis.^33^ In the MUSTANG study, the prevalence of risk factors and the cigarette smoking rate were significantly higher in CA patients than in UA patients.^10^ In a study based on the National Hospital Discharge Survey, Livingston reported that the number of CA patients per 10 000 population increased with time over the period of the study, whereas that of UA patients decreased, with the lowest portion of the curve at the mid‐1990s followed by an increasing trend.^34^

Non‐operative management for CA followed by appendectomy (IA) may be a promising treatment strategy. In the present study, the short‐term outcomes of elective surgery were superior to those of UA patients. Two meta‐analyses favored non‐operative to surgical management for CA because it was associated with a decrease in the complication and reoperation rates.^35^, ^36^ A 2017 Cochrane analysis, however, found no strong evidence in favor of non‐operative management that included IA over immediate appendectomy in CA patients.^37^ No large study showed a complication rate of IA. One systemic review, including 543 patients who underwent interval appendectomy for CA, reported that the morbidity rate is 10.4%^38^. This rate is considerably higher than the morbidity rate of EA in the present study, but it is lower than that of CA in the present study. One retrospective study showed that the morbidity rate was 5.7% (6/106)^39^, which is almost the same as our result of EA. Only a large, randomized, controlled study can resolve this complex and important clinical question.

This study had several limitations that must be addressed in future studies. First, variables not collected by the NCD could not be analyzed. For example, the NCD does not collect laboratory data or the use of antibiotics intraoperatively, postoperatively, and post‐discharge. Preoperative management of patients—including the selection and timing of antibiotic administration and whether non‐operative management was first attempted—is also unavailable for study. Use of oral antibiotics following discharge may decrease organ‐space infections in patients with CA.^40^ Second, the definition of CA in the present study is not strict because of the study’s retrospective design. The presence of a visible hole, diffuse fibrinopurulent exudate, intra‐abdominal abscess, and/or an extraluminal fecalith have been independently associated with markedly worse outcomes,^41^ but there is no unified definition of CA. The incidence of CA in the present study (14.6%) is similar to that reported in a large‐cohort study from Washington State (15.8%). Third, the EA group included not only CA patients treated with IA but patients with chronic appendicitis not treated with antibiotics preoperatively. Surgical outcomes of IA, however, may not differ from those of surgery for chronic appendicitis. Additionally, the incidence of chronic appendicitis is relatively low. Fourth, the EA group did not include patients with CA who underwent attempted IA but ultimately had emergency surgery because non‐operative management failed. Finally, we did not adjust for age or comorbidities because CA is generally found in older individuals, who have more comorbidities than those with UA.^11^

In conclusion, using high‐quality, audited, clinical data, we compared the 30‐day outcomes of CA, UA, and EA patients. We have confirmed that CA places the patient at relatively high risk, a finding similar to those reported in both a large‐cohort study and in a nationwide study. We also showed that the risk associated with EA is significantly lower than for any of the other choices. It is worth noting that the NCD data in this study were similar to those from the ACS NSQIP^11^ and the National (Nationwide) Inpatient Sample.^12^ Stratifying management based on the UA/CA status of the patient can improve appendicitis treatment outcomes, although many patients continue to have an equivocal diagnosis, which remains a challenging dilemma.

## CONFLICTS OF INTEREST

Hideki Endo, Hiroyuki Yamamoto, and Hiroaki Miyata are affiliated with the Department of Healthcare Quality Assessment at the University of Tokyo. The department is a social collaboration department supported by grants from the National Clinical Database, Johnson & Johnson KK, and Nipro Corporation.
